# *In vivo* efficacy assessment of the CDK4/6 inhibitor palbociclib and the PLK1 inhibitor volasertib in human chordoma xenografts

**DOI:** 10.3389/fonc.2022.960720

**Published:** 2022-11-25

**Authors:** Thibault Passeri, Ahmed Dahmani, Julien Masliah-Planchon, Rania El Botty, Laura Courtois, Sophie Vacher, Elisabetta Marangoni, Fariba Nemati, Sergio Roman-Roman, Homa Adle-Biassette, Hamid Mammar, Sébastien Froelich, Ivan Bièche, Didier Decaudin

**Affiliations:** ^1^ Laboratory of Preclinical Investigation, Translational Research Department, Institut Curie, University of Paris Saclay, Paris, France; ^2^ Department of Genetics, Institut Curie, University of Paris Saclay, Paris, France; ^3^ Department of Neurosurgery, Lariboisière Hospital, Assistance Publique des Hôpitaux de Paris, University of Paris, Paris, France; ^4^ Department of Translational Research, Institut Curie, University of Paris Saclay, Paris, France; ^5^ Department of Pathology, Lariboisière Hospital, Assistance Publique des Hôpitaux de Paris, University of Paris, Paris, France; ^6^ Department of Radiotherapy - Proton Therapy Center, Institut Curie, Paris-Saclay University, Orsay, France; ^7^ Department of Medical Oncology, Institut Curie, Paris, France

**Keywords:** CDKN2A/2B deletion, chordoma patient’s derived xenograft, CDK4/6 inhibitor, palbocicib, PLK1 inhibitor, volasertib

## Abstract

**Background:**

Management of advanced chordomas remains delicate considering their insensitivity to chemotherapy. Homozygous deletion of the regulatory gene *CDKN2A* has been described as the most frequent genetic alteration in chordomas and may be considered as a potential theranostic marker. Here, we evaluated the tumor efficacy of the CDK4/6 inhibitor palbociclib, as well as the PLK1 inhibitor volasertib, in three chordoma patient-derived xenograft (PDX) models to validate and identify novel therapeutic approaches.

**Methods:**

From our chordoma xenograft panel, we selected three models, two of them harboring a homozygous deletion of *CDKN2A/2B* genes, and the last one a *PBRM1* pathogenic variant (as control). For each model, we tested the palbociclib and volasertib drugs with pharmacodynamic studies together with RT-PCR and RNAseq analyses.

**Results:**

For palbociclib, we observed a significant tumor response for one of two models harboring the deletion of *CDKN2A/2B* (p = 0.02), and no significant tumor response in the *PBRM1*-mutated PDX; for volasertib, we did not observe any response in the three tested models. RT-PCR and RNAseq analyses showed a correlation between cell cycle markers and responses to palbociclib; finally, RNAseq analyses showed a natural enrichment of the oxidative phosphorylation genes (OxPhos) in the palbociclib-resistant PDX (p = 0.02).

**Conclusion:**

CDK4/6 inhibition appears as a promising strategy to manage advanced chordomas harboring a loss of *CDKN2A/2B*. However, further preclinical studies are strongly requested to confirm it and to understand acquired or *de novo* resistance to palbociclib, in the peculiar view of a targeting of the oxidative phosphorylation genes.

## Introduction

Chordomas are rare bone tumors of the skull-base and spine which originate from remnants of the notochord, explaining their common location along the neuraxis. They are characterized by a locally invasive extension despite slow growth. Considering their chemoradioresistance, a radical resection associated with a high-dose radiation is the gold-standard therapeutic strategy (1). However, despite this aggressive treatment, the disease-free survival is generally short (2, 3) due to the fact that locoregional recurrence is a common event following the initial treatment (4). The management of these tumor relapses or progression after surgery and proton beam therapy is a clinical challenge, especially for patients with metastases and inoperable recurrence disease. Difficulties in managing advanced chordomas explain the poor long-term prognosis of this cancer with an overall survival evaluated at ~30% at 10 years (3). There is, therefore, an unmet need for systemic therapies, which are especially difficult to raise given the lack of data (5–15) concerning the tumor biology (genetic driver events), and that chordomas have a relatively low mutation burden with few therapeutically actionable alterations (5, 6). Over the years, a wider use of new targeted therapies, such as tyrosine kinase inhibitors (TKI), has been developed. Unfortunately, the proportion of objective responses to all these agents was very low in chordomas (16–24). The limited number of patients included in these studies, as well as the lack of follow-up, interfere with the preliminary proof of effectiveness of these drugs, that encourages the development of several pre-clinical models (25–34).

Recently, we established and well-characterized a large panel of 12 chordoma patient-derived xenografts (PDXs) (35), including all relevant clinical features, their immunohistochemical and genomic profiles, serving as a support for preclinical drug testing. In this way, the Next Sequencing Generation (NGS) genomic characterization of our models showed a preponderance (58%) of homozygous deletions of *CDKN2A/2B* and mutations affecting the mammalian SWItch/Sucrose Non-Fermentable (SWI/SNF) chromatin remodeling complexes (such as *PBRM1*, *SMARCB1*, *ARID1A* pathogenic mutations), as described in a majority of chordomas (5, 6, 14). Mutations in the SWI/SNF complexes were also mutually exclusive with homozygous deletions of *CDKN2A/2B* in our panel. Genes encoding subunits of SWI/SNF complexes are collectively altered in over 20% of human malignancies (36) and became an interesting target for new therapies as enhancer of zeste homolog 2 (EZH2) inhibitors (35, 37, 38).

Cyclin-dependent kinase (CDK) inhibitor 2A (*CDKN2A*) is a tumor suppressor gene encoding the p16^ink4a^ protein which plays an important role in cell-cycle regulation through the inhibition of cyclin-dependent kinases (CDK) 4/6 that protects the p53 protein from degradation (39). *CDKN2A* loss or mutation is found in a wide range of malignancies and may lead to an increased CDK activity, bypassing a critical senescent signal (40). In chordomas, the regulatory gene *CDKN2A* is also frequently lost (5, 6), and thus, CDK4/6 may be in an over-activated state (41), although multiple upstream seem to be the input in the dysregulation of CDK4/6 activity (42). Palbociclib (PD 0332991), a selective inhibitor of CDK4/6, approved for the treatment of hormone-receptor positive breast cancer (43), has been recently tested in *in vitro* cell-line chordoma models harboring the loss of *CDKN2A* (41), suggesting a potential *in vivo* action of anti-CDK4/6 in chordomas harboring this genomic alteration.

The Polo-like kinase (PLK) family, particularly PLK1, has recently become an attractive oncogenic target considering its regular roles in the G2/M cell cycle checkpoint, *via* its effects on chromosome segregation, spindle assembly, and cytokinesis (44). Volasertib (BI 2536), a selective PLK1-inhibitor, showed a promising antitumor activity in other cancer preclinical models (45–47). Considering that several genomic analyses of chordoma samples reveal widespread cell cycle dysregulation (48), testing the inhibition of PLK1 in chordomas makes sense.

In this study, we therefore focused our pharmacological expertise on the CDK4/6 inhibitor palbociclib and the PLK1-inhibitor volasertib in two xenograft models harboring homozygous deletions of *CDKN2A/2B*, and in a third *PBRM1*-mutated model (control), in order to evaluate the *CDKN2A/2B* loss as a theranostic biomarker in chordomas.

## Materials and methods

### Chordoma patient-derived xenografts

Three previously established and characterized chordoma PDXs were used for *in vivo* experiments: cluster of differentiation (CD) 3, CD7, and CD39 (35). The main clinical, histological, and genomic features of our xenografts panel have been previously described (35). PDX models of chordomas were obtained by engrafting a primary tumor sample into *nude* mice (Charles River Laboratories). All *in vivo* experimental procedures, animal care, and housing were performed in accordance with the recommendations of the European Community (2010/63/UE) for the care and use of laboratory animals. Experimental procedures were specifically approved by the ethics committee of the Institut Curie CEEA-IC #118 (Authorization APAFiS# 25870-2020060410487032-v1 given by National Authority) in compliance with the international guidelines.

### Next-generation sequencing

DNA was extracted from frozen tumor samples using a standard phenol/chloroform procedure. RNA was extracted from microdissected areas using the RNeasy Mini Kit (Qiagen, Valencia, CA, USA).

DNA from xenografts and a part of patients’ tumors were then analyzed for gene mutations by targeted next-generation sequencing (NGS). The in-house NGS panel includes 571 genes of interest in oncology for diagnosis, prognosis, and theranostics including chordoma genes of interest such as *PIK3CA*, *PTEN*, *CDKN2A/2B*, *PBRM1*, *SETD2*, and *ARID1A*. The library preparation was performed using the Agilent Sureselect XT HS kit, and sequencing was completed on an Illumina NovaSeq 6000 sequencer. All variants, called using Varscan2 (v2.4.3-0), that passed the following thresholds were validated: allelic ratio above 5% and population frequency lower than 0.1% in 1000 g, ESP, or gnomAD. This large targeted NGS panel also allowed molecular analysis of tumors for CNV (copy number variation) status including *CDKN2A* homozygous deletion detection. In case of doubt concerning the originating link between the patient’s tumors and PDXs, an identity monitoring was performed based on polymorphisms.

### Antitumor efficacy of targeted therapy drugs

Considering the results of the molecular analysis of our chordoma xenograft panel, two drugs on three PDXs (CD3, CD7, and CD39) were considered in our pharmacological program, i.e. the CDK4/6 inhibitor palbociclib (Ibrance^®^, Pfizer) and the PLK1 inhibitor volasertib (Boehringer Ingelheim). This last targeted treatment was tested in the field of our huge expertise developed in various types of human cancers, such as non-small cell lung cancers (46) and breast cancers (47). Palbociclib was administered orally at a dose of 75 mg/kg/day (tween 80 0.5% + methylcellulose 0.5% + water 99%), once daily, 5 days per week; volasertib was administered orally at the dose of 10 mg/kg/day (methylcellulose 0.5% + tween 80 0.5% + DMSO 2% + water 97%), once daily, 4 days per week. The treatment was administered from day 1 to mouse sacrifice. Drugs were sourced from the oncological department of Institut Curie, Paris, France.

For *in vivo* therapeutic studies, a 15 mm^3^ tumor fragment was grafted into 30 female immunodeficiency *nude* mice. Mice bearing growing tumors with a volume of 60–150 mm^3^ were randomly assigned to control or treatment groups. Animals with tumor volumes outside this range were excluded. Mice were weighted and tumors were measured once a week. Considering the slow growth of these tumors, xenografted mice were sacrificed when tumor volume reached a mean volume of 500 mm^3^.

Tumor volumes were calculated using two perpendicular diameters with calipers as follows: *V (volume) = (a×b)^2^/2* where *a* and *b* are the largest and smallest perpendicular tumor diameters (in mm). Relative tumor volumes (RTV) were calculated from the following formula: RTV = (V*_x_*/V1), where V*_x_* is the tumor volume on day x and V1 is the tumor volume at initiation of therapy (day 1). Antitumor activity was evaluated according to tumor growth inhibition (TGI), calculated according to the following formula: percent GI = 100 − (RTVt/RTVc ×100), where RTVt is the median RTV of treated mice and RTVc is the median RTV of controls, both at a given time point when the antitumor effect was optimal. A meaningful biological effect was defined as a TGI of at least 50% (49). The statistical significance of differences observed between the individual RTVs corresponding to the treated mice and control groups was calculated using the two-tailed Mann–Whitney *U* test.

Moreover, to evaluate the response to treatments observed in the three models according to individual mouse variability, we decided to consider each mouse as one tumor-bearing entity. Hence, in all *in vivo* experiments, a relative tumor variation (RTVV)) was calculated for each treated mouse as follows: [(RTVt/mRTVc)], where RTVt is the relative tumor volume of the treated mouse and mRTVc is the median relative tumor volume of the corresponding control group at the end of treatment. We then calculated an overall response rate (ORR) for each treated mouse as follows: ORR = [(RTVV) - 1]. A tumor was considered to be responding to treatment, if the ORR was below -0.5. The statistical significance of the ORR between tested treatments was determined using Fisher’s Exact test. Finally, we evaluated the impact of treatments on tumor progression, by evaluating all progression-free survival probabilities taking tumor doubling time (RTV × 2) and time for RTV × 4 into account. A log-rank (Mantel–Cox) test was used to perform treatment comparisons.

### Pharmacodynamics study by RT-PCR analysis and RNAseq after *in vivo* pharmacological experiments

A RT-PCR study was performed after the administration of palbociclib and volasertib drugs on the two models CD3 and CD7. The RT-PCR technique has been previously described by our group (50). Results, expressed as N-fold differences in target gene expressions relative to the murine and human *TBP* genes (both the murine and the human TBP transcripts), and termed N_target_, were determined as N_target_ = 2^ΔCtsample^, where the ΔCt value of the sample was determined by subtracting the Ct value of the specific target gene from the Ct value of the TBP. The N_target_ values of the samples were subsequently normalized so that the value for “basal mRNA level” (smallest amount of quantifiable target gene mRNA, Ct = 35) was 1 for selected genes. The expression of the following cancer genes potentially implied in palbociclib resistance (51, 52) was studied: *CCNE1*, *CDK4*, *PTEN*, *RB1*, and *E2F1*. The *PLK1* gene was also studied, as the target of PLK1 inhibitors. Finally, we studied some genes implied in the proliferation, or overexpressed in chordomas: MKI67, *MYC*, *TBXT* and *EGFR (* 53). The nucleotide sequences of the gene primers used are available on request.

For RNAseq analyses, RNA was extracted from microdissected areas using the RNeasy Mini Kit (Qiagen, Valencia, CA, USA). Library preparation was performed using the QuantSeq 3′ mRNA-Seq reverse (REV) Library Prep Kit (Lexogen, Vienna, Austria) using 150 ng of total RNA, and according to the manufacturer’s instruction. The pool was sequenced on a NovaSeq 6000 SP 2x75bp flow cell (Illumina Inc., San Diego, CA, USA). RNA sequencing data were analyzed using the BlueBee Genomics Platform (Lexogen, Vienna, Austria). Gene Ontology Enrichment Analysis was performed using the ShinyGO (http://bioinformatics.sdstate.edu/go/) and gProfiler (https://biit.cs.ut.ee/gprofiler/gost) Web sites.

### Statistical analyses

Statistical analyses were performed using the Prism v9.0 software (GraphPad Software, Inc., La Jolla, CA, USA). Statistical characteristics were used to describe all variables. Numerical variables were expressed as the median or mean and standard deviation, as appropriate. Categorical variables were expressed as the count and percentage. Variables were tested by the Mann-Whitney *U* test, Fisher’s Exact test, or chi-squared test, as appropriate. Survival distributions were determined using the Kaplan–Meier method and the log-rank (Mantel–Cox) test was used to compare groups. Every statistical test used in this manuscript was two-tailed, and p-values less than 0.05 were considered as significant.

## Results

### Patient-derived xenograft selection

**Figure 1:**
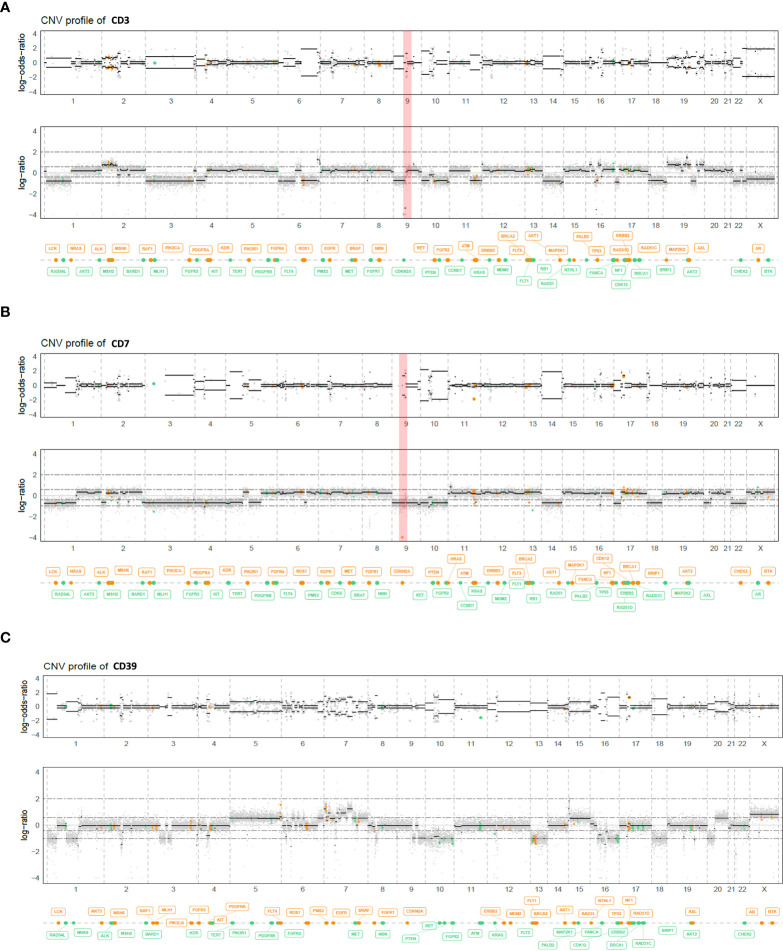
Genome view profiles of the allele-specific copy number of the CD3 **(A)**, CD7 **(B)**, and CD39 **(C)** chordoma PDX models. The top graph log-odds-ratio represents the B-Allele Frequency (BAF), and the bottom graph the log depth ratio between tumor and a healthy witness. Both CD3 and CD7 chordoma PDX models harbored a homozygous deletion of *CDKN2A/2B* (red bar), whereas CD39 did not.

The histological and genomic features of the three PDXs are described in **Table 1**. CD3 was obtained from a patient’s sacral primary tumor previously operated on and displaying a homozygous deletion of *CDKN2A/2B;* CD7 was obtained from a skull base primary tumor previously operated on and irradiated and displaying a homozygous deletion of *CDKN2A*/*2B;* CD39 was obtained from a skull base primary tumor previously operated on and presenting a pathogenic *PBRM1* variant (c.599C>G), without *CDKN2A*/*2B* deletion (**Figure 1**). The CD39 model which does not harbor any loss-of-function genomic alteration of *CDKN2A* or *CDKN2B* but a *PBRM1*-variant was chosen as a negative control in order to reinforce the fact that *CDKN2A/2B* might be a biomarker of response to palbociclib. None of these models harbored another CNV or pathogenic variant of theranostic interest.

**Table 1:** Histopathological and molecular features of the three selected PDX models.

Models	Histology type*	Molecular alteration	Targeted-therapy
CD3	Conventional	*CDKN2A/2B* loss	Palbociclib Volasertib
CD7	Conventional	*CDKN2A/2B* loss	Palbociclib Volasertib
CD39	Conventional	*PBRM1* variant (c.599C>G; p.(Ser200Ter))	Palbociclib Volasertib

*According to the WHO (World Health Organization) 2020.

CD, cluster of differentiation.

### *In vivo* antitumor efficacy of targeted therapies

*In vivo* therapeutic experiments with palbociclib and volasertib were done on these three xenografts. For each model, PDX tumor-bearing mice were randomized into treatment and control groups (n = 4–7 mice per group). For the palbociclib drug, TGI was calculated at 80, 49, and 85 days, for CD3, CD7, and CD39, respectively. For the volasertib drug, TGI was calculated at 77, 28, and 51 days, for CD3, CD7, and CD39, respectively.

**Figure 2:**
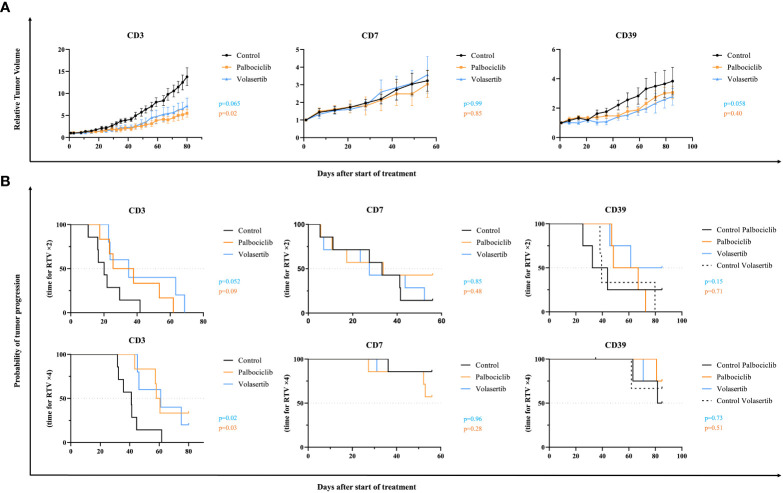
*In vivo* efficacy of palbociclib and volasertib, in the CD3, CD7, and CD39 chordoma PDXs. PDX tumor-bearing mice were randomized into each treatment group (n = 4–7 mice per group), and treated with palbociclib 75 mg/kg, 5 days per week (orange), or volasertib 10 mg/kg, 4 days per week (blue). Untreated control is shown in black. **(A)** Relative tumor volumes. Tumor growth was evaluated by plotting the mean of the relative tumor volume ± SD per group. **(B)** Probability of tumor progression. The time to reach RTV × 2 and RTV × 4 for each treated mouse has been calculated using Kaplan Meier curves and log-rank test.

*In vivo* responses to palbociclib and volasertib are shown in the **Figure 2**. For the CD3 xenograft, palbociclib induced a significant TGI (p = 0.02) with an optimal TGI of 60%, whereas volasertib showed a slight tumor growth response close to statistical significance (p = 0.065) (**Figure 2A**). The probability of progression-free survival at RTV × 4 was significantly longer in the palbociclib and volasertib treatments groups than in the control (p = 0.03 and p = 0.02, respectively), but not at RTV× 2 (p = 0.09 and p = 0.052, respectively) (**Figure 2B**).

For the CD7 xenograft, we did not observe any tumor growth response to both palbociclib and volasertib (p = 0.85 and p > 0.99, respectively), as well as for the CD39 xenograft without *CDKN2A/2B* deletion (p = 0.40 and 0.058, respectively).

**Figure 3:**
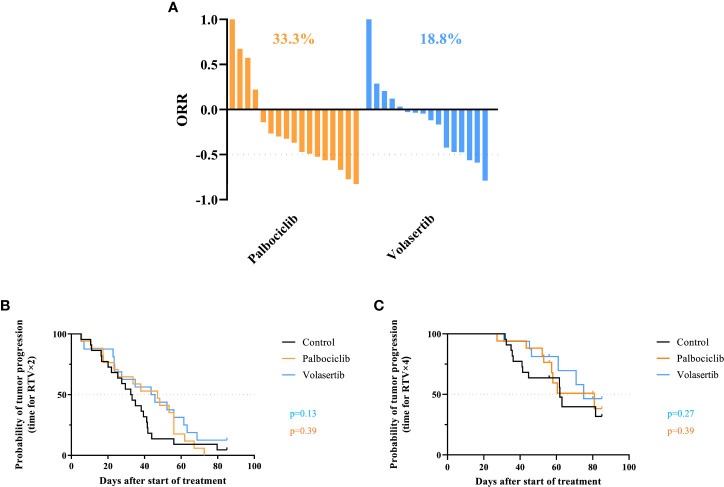
Individual tumor responses in the three treated models. **(A)** Overall response rate (ORR) in all treated chordoma PDXs after palbociclib (n = 17) and volasertib (n = 16). A tumor was considered to be responding to treatment if ORR was below -0.5. **(B, C)** Probability of progression (RTV × 2 and RTV × 4) in all treated chordoma PDXs after palbociclib and volasertib treatments.

Hence, when we looked at the individual tumor responses in the three treated models (**Figure 3A**), the ORRs lower than -0.5 were 33.3% and 18.8% after palbociclib and volasertib, respectively (p = 0.25). Finally, the study of the progression-free survival across the three PDX models at RTV × 2 and RTV × 4 did not show any significant effect for palbociclib (p = 0.39 and p = 0.39 for RTV × 2 and RTV × 4, respectively) nor with volasertib (p=0.13 and p=0.27 for RTV x 2 and RTV x 4 respectively) (**Figures 3B, C**).

### Pharmacodynamics study

**Figure 4:**
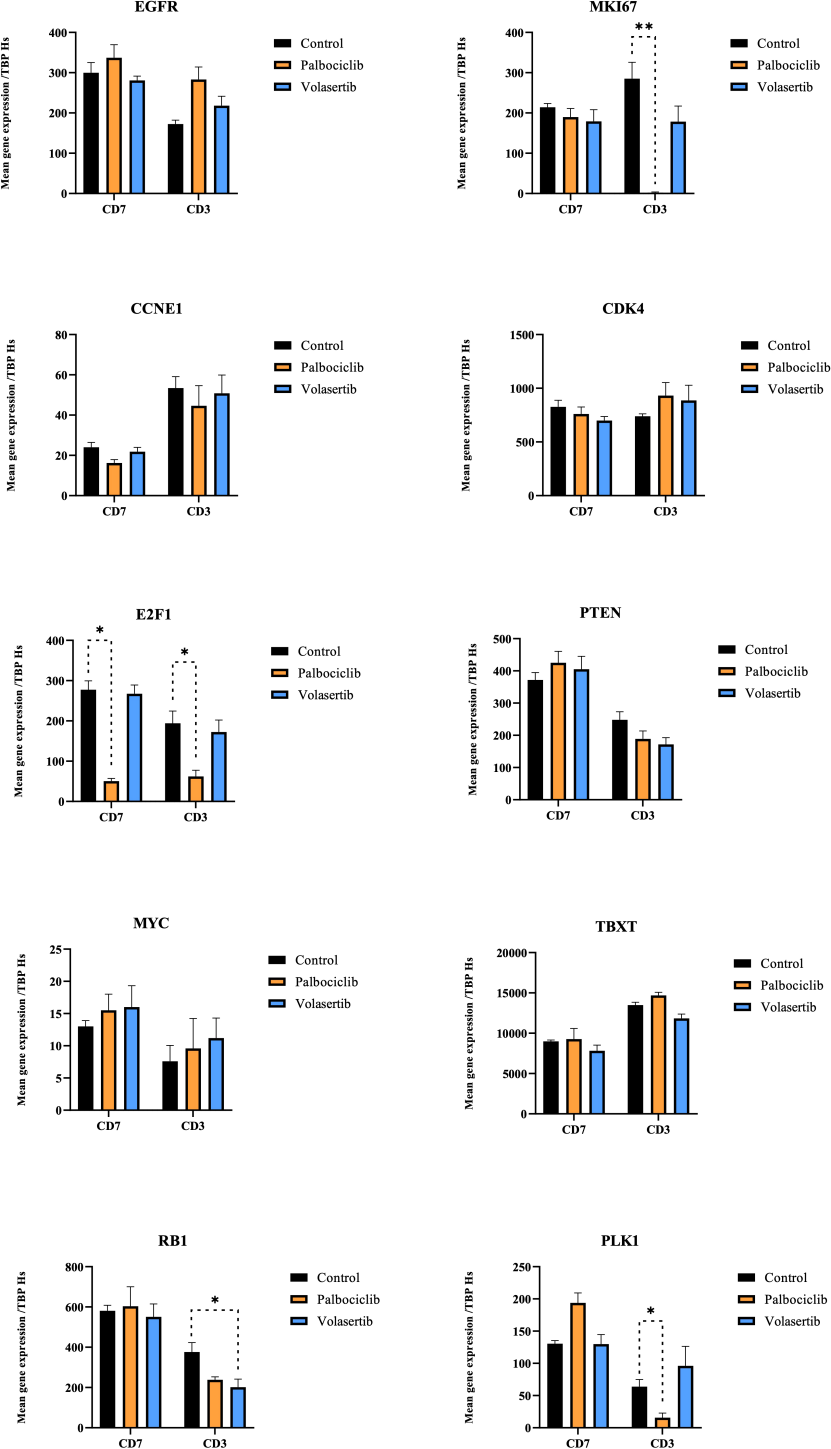
Gene expression modifications after treatments with palbociclib and volasertib (CD3 and CD7 models). Quantitative RT-PCR was performed to measure the expression of the target genes. Data are presented as level of gene expression compared to the standard level of *Homo sapien* (Hs) TBP. Genes expressions were compared between mice treated with the indicated agent and the control group. Error bars show the standard error of the mean. * and ** achieve statistical significance compared to control (p < 0.05 and p < 0.01, respectively) by Mann–Whitney *U* test.

Using RT-PCR analyses, we first looked at modifications of candidate gene expression occurring after palbociclib and volasertib *in vivo* therapies in CD3 and CD7 models compared to the control groups (**Figure 4**). For that, we applied RT-qPCR on the genes of interest (described in the materials and methods section) on 28 samples assigned to the control or treatment group. After palbociclib treatment, we observed a significant decrease in human *MKI67* (p = 0.008), *E2F1* (p = 0.016), and *PLK1* (p = 0.016) gene expression in the *in vivo* responding CD3 xenograft; in contrast, we only observed decreased gene expression in *E2F1* (p = 0.029) in the non-responding model CD7. No significant modification of gene expression was noted for *CDK4* (palbociclib target), *MYC*, *TBXT*, *EGFR*, *CCNE1*, and *RB1* in both studied models. After volasertib administration, we observed a significant gene expression modification of *RB1* (p = 0.03) in the CD3 model. No significant modification of gene expression was noted for the other genes, including *PLK1* (volasertib target).

**Figure 5:**
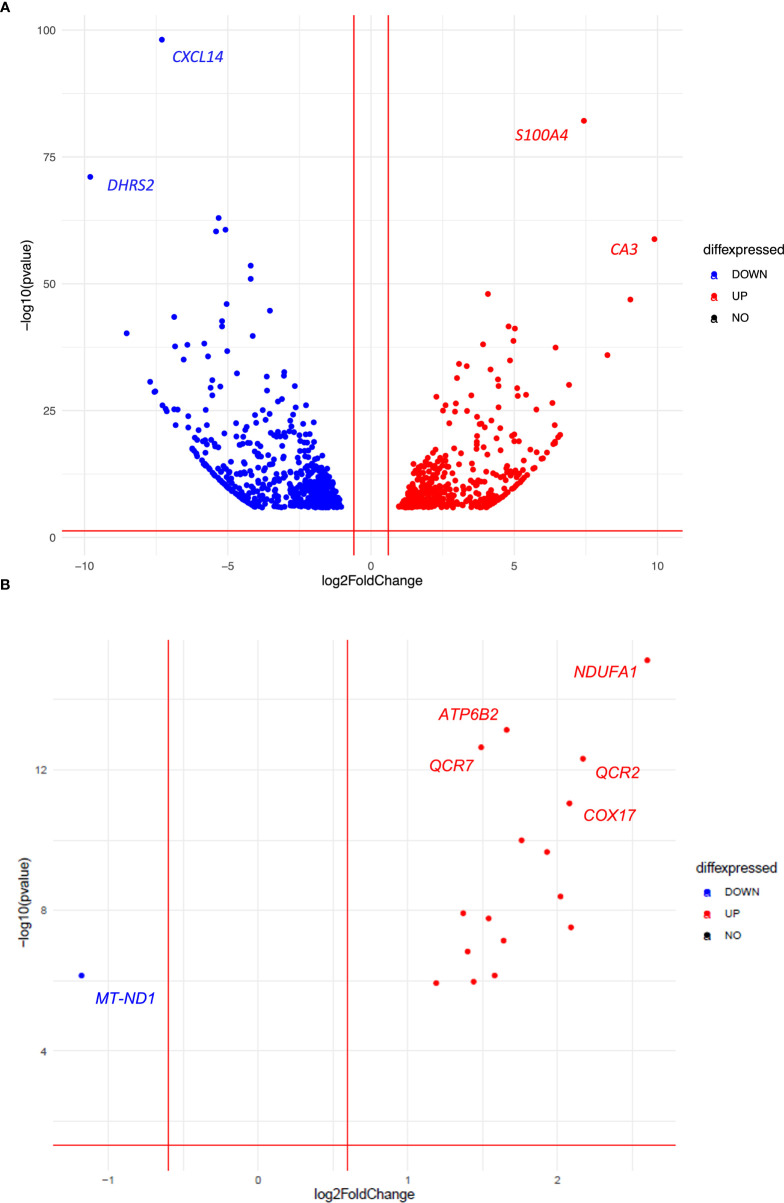
Volcanoplots illustrating the top 1000 genes **(A)** and all genes related to the oxidative phosphorylation (OxPhos) pathway **(B)**, in which we observed a significant gene expression variation in both untreated CD3 and CD7 PDX models (adjusted p-value).

To better understand the opposite efficacy of palbociclib in the two *CDKN2A/2B* deleted CD3 and CD7 PDXs, we then performed RNAseq analyses in both control and treated groups (three tumors per group). In a differential supervised analysis, comparing control tumors and tumors treated with palbociclib in both PDX models, we observed a more important modification of gene expression in the responding CD3 PDX model (n = 318 genes, **Supplementary Table S1**) compared to the non-responding CD7 PDX model (n = 12 genes; **Supplementary Table S2**), which correlated with *in vivo* observations. Next, we tested the enrichment of the set of genes resulting from the KEGG database. As expected, we found a significant decrease in the enrichment of cell cycle genes in the responding CD3 tumors (p = 0.04), such as *TOP2A* (p = 2.2 e^-26^), *MKI67* (p = 4.3 e^-20^), and *AURKB* (2.5 e^-8^). Moreover, we compared in a supervised analysis the gene expression of control untreated CD3 PDX tumors and control untreated CD7 PDX tumors in order to find predictive biomarkers of intrinsic palbociclib resistance (**Supplementary Table S3**). We observed an upregulation of genes implied in hypoxia such as *S100A4* (p = 6.5 e^-79^) and *CA3* (p = 3.8 e^-56^) in the resistant CD7 PDX model (**Figure 5A**). Among the top 1000 genes in which we observed a significant gene expression variation in both the models (adjusted p-value), we noted an enrichment of the oxidative phosphorylation genes in the resistant CD7 PDX model (p = 0.02) using the KEGG data base, such as *NDUFA1* (p = 6.5 e^-14^), *NDUFB2* (p = 1.2 e^-07^), *COX6C* (p = 4.4 e^-07^), *COX7A2* (p = 1.9 e^-05^), *COX7C* (p = 3.2 e^-7^), and *COX17* (p = 4.2 e^-10^) (**Figure 5B**). To resume the pharmacodynamic study, we observed a decrease in the expression of genes implied in the cell cycle in the responding CD3 compared to the CD7 PDX model treated by palbociclib. Moreover, we noted a significant enrichment of the expression of genes implied in oxidative phosphorylation (OxPhos) into the non-responding control CD7 PDX model in comparison with the responding control CD3 PDX model.

## Discussion

In this current study, we explored the antitumor effect of the targeted therapy palbociclib in three PDX chordoma models in order to consider the homozygous deletion of *CDKN2A/2B* as a theranostic biomarker of response to palbociclib, as suggested in other cancer types (43, 54, 55). We observed various responses to palbociclib concerning both models (CD3, CD7) harboring a loss of *CDKN2A/2B*, and no tumor efficacy in the non-*CDKN2A/2B*-deleted *PBRM1*-mutated PDX (CD39). We also tested the PLK1 inhibitor volasertib in these three PDXs and did not observe any significant anti-tumor response.

**Figure 6:**
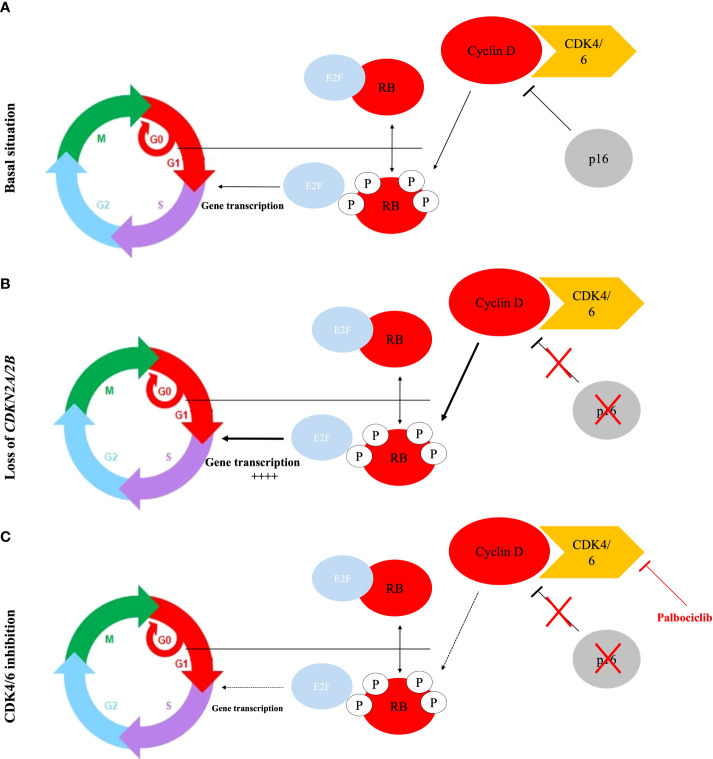
The classic model for the regulation of the G1/S transition by cyclins and CDK. **(A)** In basal situation, RB protein phosphorylation performed by cyclin D-CDK4/6 kinase induces E2F releasing and facilitates the expression of E2F target genes, which are critical for initiation of DNA synthesis and entry into S-phase. CDK4/6 activity is repressed by the p16 protein. **(B)** Loss of p16/CDKN2A/2B activity leads to an overactivation of the CDK4/6 activity and therefore an uncontrolled cell proliferation. **(C)** The CDK4/6 inhibitor re-establishes the activity of the RB protein activity as an inhibitor of cell division.

Chordoma tumors are known to overexpress multiple kinases (56) (included PDGFR-α, PDGFR-β, c-Kit, c-Met, pAKT, mTOR, HER2, VEGFR, and EGFR) which are involved in a multitude of cellular functions relevant to cancer pathogenesis. These kinases are well-studied in the field of oncology, and several FDA-approved drugs on the market targeting each kinase can be used as repurposing candidates for advanced chordomas. The first pre- and clinical results of some FDA-approved drugs targeting EGFR, PDGFR-α, PDGFR-β, and c-Kit, have shown partial objective response and clinical benefit in chordomas (57). The lack of data concerning the biology of chordomas (genetic driver events) and theranostic markers hampers the selection of molecularly guided therapies and may explain these sparse results. As kinases, CDK4 and CDK6 play a crucial role in the G1-S phase transition, which is an important restriction point in the normal cell cycle, often reduced in cancer, leading to uncontrolled cell proliferation (**Figure 6**). Importantly, in chordomas, as well as in our PDX chordoma panel (35), the CDK4/6 regulatory gene *CDKN2A* (also known as p16) is frequently lost (5, 6), leading to the fact that CDK4/6 may be found in an over-activated state (41). However, it should be noted that other signaling pathways, in addition to *CDKN2A* loss, are inputted in cyclin D overexpression and promote CDK4/6 activity, leading to uncontrolled cell proliferation (42). Palbociclib, a specific inhibitor of CDK4/6, has recently been tested in *in vitro* cell line chordoma models harboring the loss of *CDKN2A* and showed an efficient inhibition of the tumor cell growth (41). Finally, the efficacy of palbociclib has been tested in numerous clinical trials for cancers, and is also currently being tested in a phase II trial, in patients with locally advanced/metastatic chordomas (NCT03110744).

In our *in vivo* pharmacological program, the CD3 model harboring *CDKN2A/2B* showed a significant tumor growth inhibition (p = 0.02), while the CD7 model showed no tumor response (p = 0.85). Pharmacodynamic study (RT PCR and RNAseq) confirmed the difference between both PDX models harboring both the loss of *CDKN2/2B*. Indeed, in the responding CD3 PDX model, we observed a decrease in the expression of genes implied in the cell cycle, such as *MKI67* (p = 0.008), *E2F1* (p = 0.016)*, PLK1* (p = 0.016), *TOP2A* (p = 2.2 e^-26^), and *AURKB* (p = 2.5 e^-8^), compared to the non-responding model (CD7). We did not observe tumor response to palbociclib with the xenograft CD39 (p = 0.40) (which did not harbor a loss of *CDKN2A/2B)*; such an observation raises the possibility to consider *CDKN2A/2B* loss as a theranostic marker of palbociclib antitumor efficacy in chordomas. Considering the various sensitivities to palbociclib, we can formulate the hypothesis on which tumor heterogeneity and preexistence of a resistant clonal subtype to palbociclib in the CD7 PDX model might be present. Indeed, chordoma is known as a heterogeneous tumor (58) with various cell phenotypes. Genetic heterogeneity contributes to selecting a clonal cell population during tumor development and progression, and is well-known as a prominent contributor to therapeutic failure (59). This hypothesis is reinforced by genetic analyses performed on the CD7 chordoma PDX model, which confirmed the loss of *CDKN2A/2B* in treated and control mice, but not in the primary patient’s tumor (35), suggesting a spatial tumor heterogeneity in which some cells harbor the homozygous deletion of *CDKN2A*, and others do not. Moreover, as observed in hormone receptor-positive breast cancers, many tumors demonstrate *de novo* (or intrinsic) resistance to palbociclib, implying genomic alterations (60). When we compared the gene expression in the RNAseq-supervised analysis in non-treated CD3 and CD7 PDX tumors, we observed a significant enrichment of the expression of genes implied in oxidative phosphorylation (OxPhos) into the non-responding CD7 PDX model, as compared to the responding CD3 PDX model. Metabolism reprogramming is well known as a hallmark of cancer (61). While normal cells commonly use mitochondrial oxidative metabolism for energy generation, tumor cells often switch to aerobic glycolysis, described as the Warburg effect (62). Classically, glycolytic metabolism is involved in the induction of acquired drug resistance, such as in estrogen-receptor breast cancer treated with palbociclib (63). However, some authors also suggested that drug resistance could be associated with an upregulation of the oxidative phosphorylation (OxPhos) pathway, which may be explained by the “reverse Warburg effect” (64, 65). Evans et al. (66) recently showed an OxPhos dependence in chemotherapy-resistant triple-negative breast cancer. Moreover, radiation therapy seems to increase *in vitro* and *in vivo* OxPhos activity (67). Thus, we can suppose a selection of clonal subtypes to palbociclib in the CD7 PDX model, increased by the history of radiation, leading to a higher OxPhos activity in this model. Interestingly, drug resistance can be reversed by blocking OxPhos (68). In several cancers, new OxPhos inhibitors have already been identified, and are under preclinical and/or clinical evaluation (66, 69). Moreover, favorable results about the synergistic antitumoral effect of the combination of OxPhos inhibitors and palbociclib in a triple-negative breast cancer cell line (66), encourage testing the use of this drug combination or OxPhos inhibitors alone in palbociclib-resistant PDX chordoma models harboring the *CDKN2A/2B* loss. However, some authors revealed an upregulation of oxidative metabolism (OxPhos) in CDK4/6 inhibitor-tolerant uveal melanomas (70), suggesting further experimental research to conclude a causal association in the Oxphos dependency of palbociclib-resistant chordomas. Although being not fully understood, several other resistance mechanisms of CD4/6 inhibitors exist, as mechanisms bypassing the CDK4/6 inhibition *via* activating CDK2 signaling, which is another way that phosphorylates RB (52).

The PLK1 inhibitor showed a slight antitumor efficacy without significant activity (p = 0.065) in one model (CD3). These results could be explained by the absence of concomitant amplification or copy number gains of *CCND1* and *CCNE2* in our chordoma PDXs models (35), both of which are involved in RB inactivation driving tumor cells through the S phase (47). PLK1 inhibition seems to be more promising in a combination of treatments than in monotherapy (46, 71), therefore suggesting the importance of future preclinical trials in order to assess the combination of PLK1 inhibitors with other targeted drugs such as palbociclib. Indeed, PLK1 inhibition results in a G2-M arrest (72). Consequently, the combination of drugs resulting in a dual G1 (palbociclib) and G2-M arrest (volasertib) might therefore be addressed in chordomas.

### Study limitations

Considering our disparate results and the low number of tested models (n = 3), there is a lack of support evidence to consider *CDKN2A/2B* loss as a potential biomarker for CDK4/6 inhibitor sensitivity, suggesting the importance of further studies on more preclinical models. Secondly, we did not perform drug combinations, especially with volasertib and palbociclib, considering the slow-growth of the tumor in PDX models (35). Moreover, we also hypothesized that the enrichment of the expression of genes implied in oxidative phosphorylation (OxPhos) could lead to primary resistance in chordoma. However, our results are insufficient to definitely conclude a causal association considering the few tested models and the absence of additional pharmacogenomic experiments.

## Conclusion

Considering the frequency of genomic alterations affecting the *CDKN2A/2B* gene in chordomas, targeting this biomarker was fundamental. The present study described for the first time the *in vivo* activity of a CDK4/6 inhibitor and a PLK1 inhibitor in two human chordoma xenografts harboring homozygous deletions of *CDKN2A/2B.* We also hypothesized that OxPhos activity could lead to as a resistance mechanism to CDK4/6 inhibitors in chordomas. CDK4/6 inhibition may be effective, but further studies in several PDX models are strongly necessary to confirm such an observation, and to identify predictive markers of response or resistance to palbociclib in chordomas and reverse primary or secondary resistances. Investigating volasertib in combination with palbociclib could also provide a novel therapeutic strategy.

## Data availability statement

The data presented in the study are deposited in the GEO repository, accession number GSE216419 (https://www.ncbi.nlm.nih.gov/geo/query/acc.cgi?acc=GSE216418).

## Ethics statement

The animal study was reviewed and approved by Ethics committee of the Institut Curie CEEA-IC #118 (Authorization APAFiS# 25870-2020060410487032-v1 given by National Authority).

## Author contributions

Conceptualization, TP, JM-P, HM, SF, IB and DD. Data curation, TP. Formal analysis, TP. Investigation, TP. Methodology, TP and DD. Project administration, DD. Resources, TP, AD, LC, RB, FN, SV, HA-B. Software, TP. Supervision, JMP, HAB, HM, SF, IB and DD. Validation, SR-R, EM, HM, SF, JM-P and IB. Visualization, TP. Writing – original draft, TP and DD. Writing – review and editing, JM-P and IB. All authors contributed to the article and approved the submitted version.

## Acknowledgments

We wish to thank the Animal Platform of the Institut Curie and Raphaëlle Galy for English language editing.

## Conflict of interest

The authors declare that the research was conducted in the absence of any commercial or financial relationships that could be construed as a potential conflict of interest.

## Publisher’s note

All claims expressed in this article are solely those of the authors and do not necessarily represent those of their affiliated organizations, or those of the publisher, the editors and the reviewers. Any product that may be evaluated in this article, or claim that may be made by its manufacturer, is not guaranteed or endorsed by the publisher.

## Supplementary material

Supplementary Table 1 Differential supervised analysis of the gene expression, comparing control tumors and tumors treated with palbociclib for the responding CD3 PDX model.

Supplementary Table 2 Differential supervised analysis of the gene expression, comparing control tumors and tumors treated with palbociclib for the non-responding CD7 PDX model.

Supplementary Table 3 Differential supervised analysis of the gene expression of control CD3 PDX tumors and control CD7 PDX tumors control PDX tumors.

## References

[1] Stacchiotti S Sommer J. Chordoma global consensus group. building a global consensus approach to chordoma: A position paper from the medical and patient community. Lancet Oncol (2015) 16:e71–83. doi: 10.1016/S1470-2045(14)71190-8 25638683

[2] Zou M-X Lv G-H Zhang Q-S Wang S-F Li J Wang X-B. Prognostic factors in skull base chordoma: A systematic literature review and meta-analysis. World Neurosurg (2018) 109:307–27. doi: 10.1016/j.wneu.2017.10.010 29045855

[3] Bohman L-E Koch M Bailey RL Alonso-Basanta M Lee JYK. Skull base chordoma and chondrosarcoma: Influence of clinical and demographic factors on prognosis: a SEER analysis. World Neurosurg (2014) 82:806–14. doi: 10.1016/j.wneu.2014.07.005 25009165

[4] Stacchiotti S Gronchi A Fossati P Akiyama T Alapetite C Baumann M. Best practices for the management of local-regional recurrent chordoma: A position paper by the chordoma global consensus group. Ann Oncol (2017) 28:1230–42. doi: 10.1093/annonc/mdx054 PMC5452071 28184416

[5] Tarpey PS Behjati S Young MD Martincorena I Alexandrov LB Farndon SJ. The driver landscape of sporadic chordoma. Nat Commun (2017) 8. doi: 10.1038/s41467-017-01026-0 PMC5638846 29026114

[6] Bai J Shi J Li C Wang S Zhang T Hua X. Whole genome sequencing of skull-base chordoma reveals genomic alterations associated with recurrence and chordoma-specific survival. Nat Commun (2021) 12:757. doi: 10.1038/s41467-021-21026-5 33536423 PMC7859411

[7] Le LP Nielsen GP Rosenberg AE Thomas D Batten JM Deshpande V. Recurrent chromosomal copy number alterations in sporadic chordomas. PloS One (2011) 6:e18846. doi: 10.1371/journal.pone.0018846 21602918 PMC3094331

[8] Duan Z Feng Y Shen J Hornicek F. Genomic and epigenetic instability in chordoma: Current insights. AGG (2014) 67. doi: 10.2147/AGG.S50523

[9] Wang L Zehir A Nafa K Zhou N Berger MF Casanova J. Genomic aberrations frequently alter chromatin regulatory genes in chordoma. Genes Chromosomes Cancer (2016) 55:591–600. doi: 10.1002/gcc.22362 27072194 PMC5031498

[10] Diaz RJ Guduk M Romagnuolo R Smith CA Northcott P Shih D. High-resolution whole-genome analysis of skull base chordomas implicates FHIT loss in chordoma pathogenesis. Neoplasia (2012) 14:788–98. doi: 10.1593/neo.12526 PMC3459274 23019410

[11] Mattox AK Yang B Douville C Lo S-F Sciubba D Wolinsky JP. The mutational landscape of spinal chordomas and their sensitive detection using circulating tumor DNA. Neurooncol Adv (2021) 3:vdaa173. doi: 10.1093/noajnl/vdaa173 33543146 PMC7850091

[12] Cottone L Eden N Usher I Lombard P Ye H Ligammari L. Frequent alterations in p16/CDKN2A identified by immunohistochemistry and FISH in chordoma. J Pathol Clin Res (2020) 6:113–23. doi: 10.1002/cjp2.156 PMC7164370 31916407

[13] Zuccato JA Patil V Mansouri S Liu JC Nassiri F Mamatjan Y. DNA Methylation-based prognostic subtypes of chordoma tumors in tissue and plasma. Neuro-Oncology (2022) 24:442–54. doi: 10.1093/neuonc/noab235 PMC8917394 34614192

[14] Yang C Sun J Yong L Liang C Liu T Xu Y. Deficiency of PTEN and CDKN2A tumor-suppressor genes in conventional and chondroid chordomas: Molecular characteristics and clinical relevance. Onco Targets Ther (2020) 13:4649–63. doi: 10.2147/OTT.S252990 PMC7259488 32547095

[15] Choy E MacConaill LE Cote GM Le LP Shen JK Nielsen GP. Genotyping cancer-associated genes in chordoma identifies mutations in oncogenes and areas of chromosomal loss involving CDKN2A, PTEN, and SMARCB1. PloS One (2014) 9:e101283. doi: 10.1371/journal.pone.0101283 24983247 PMC4077728

[16] Singhal N Kotasek D Parnis FX. Response to erlotinib in a patient with treatment refractory chordoma. Anticancer Drugs (2009) 20:953–5. doi: 10.1097/CAD.0b013e328330c7f0 19730087

[17] Stacchiotti S Marrari A Tamborini E Palassini E Virdis E Messina A. Response to imatinib plus sirolimus in advanced chordoma. Ann Oncol (2009) 20:1886–94. doi: 10.1093/annonc/mdp210 19570961

[18] Lindèn O Stenberg L Kjellén E. Regression of cervical spinal cord compression in a patient with chordoma following treatment with cetuximab and gefitinib. Acta Oncol (2009) 48:158–9. doi: 10.1080/02841860802266672 18752082

[19] Lebellec L Chauffert B Blay J-Y Le Cesne A Chevreau C Bompas E. Advanced chordoma treated by first-line molecular targeted therapies: Outcomes and prognostic factors. a retrospective study of the French sarcoma group (GSF/GETO) and the association des neuro-oncologues d’Expression française (ANOCEF). Eur J Cancer (2017) 79:119–28. doi: 10.1016/j.ejca.2017.03.037 28478340

[20] Stacchiotti S Morosi C Lo Vullo S Casale A Palassini E Frezza AM. Imatinib and everolimus in patients with progressing advanced chordoma: A phase 2 clinical study. Cancer (2018) 124:4056–63. doi: 10.1002/cncr.31685 30216418

[21] Hindi N Casali PG Morosi C Messina A Palassini E Pilotti S. Imatinib in advanced chordoma: A retrospective case series analysis. Eur J Cancer (2015) 51:2609–14. doi: 10.1016/j.ejca.2015.07.038 26283036

[22] George S Merriam P Maki RG Van den Abbeele AD Yap JT Akhurst T. Multicenter phase II trial of sunitinib in the treatment of nongastrointestinal stromal tumor sarcomas. J Clin Oncol (2009) 27:3154–60. doi: 10.1200/JCO.2008.20.9890 PMC2716937 19451429

[23] Stacchiotti S Longhi A Ferraresi V Grignani G Comandone A Stupp R. Phase II study of imatinib in advanced chordoma. J Clin Oncol (2012) 30:914–20. doi: 10.1200/JCO.2011.35.3656 22331945

[24] Stacchiotti S Tamborini E Lo Vullo S Bozzi F Messina A Morosi C. Phase II study on lapatinib in advanced EGFR-positive chordoma. Ann Oncol (2013) 24:1931–6. doi: 10.1093/annonc/mdt117 23559153

[25] Brüderlein S Sommer JB Meltzer PS Li S Osada T Ng D. Molecular characterization of putative chordoma cell lines. Sarcoma (2010) 2010:630129. doi: 10.1155/2010/630129 21253487 PMC3022207

[26] Liu X Nielsen GP Rosenberg AE Waterman PR Yang W Choy E. Establishment and characterization of a novel chordoma cell line: CH22. J Orthop Res (2012) 30:1666–73. doi: 10.1002/jor.22113 22504929

[27] Scheil S Brüderlein S Liehr T Starke H Herms J Schulte M. Genome-wide analysis of sixteen chordomas by comparative genomic hybridization and cytogenetics of the first human chordoma cell line, U-CH1. Genes Chromosomes Cancer (2001) 32:203–11. doi: 10.1002/gcc.1184 11579460

[28] Bosotti R Magnaghi P Di Bella S Cozzi L Cusi C Bozzi F. Establishment and genomic characterization of the new chordoma cell line chor-IN-1. Sci Rep (2017) 7:9226. doi: 10.1038/s41598-017-10044-3 28835717 PMC5569021

[29] Rinner B Froehlich EV Buerger K Knausz H Lohberger B Scheipl S. Establishment and detailed functional and molecular genetic characterisation of a novel sacral chordoma cell line, MUG-Chor1. Int J Oncol (2012) 40:443–51. doi: 10.3892/ijo.2011.1235 22002331

[30] Hsu W Mohyeldin A Shah SR, a Rhys CM Johnson LF Sedora-Roman NI. Generation of chordoma cell line JHC7 and the identification of brachyury as a novel molecular target. J Neurosurg (2011) 115:760–9. doi: 10.3171/2011.5.JNS11185 PMC4273567 21699479

[31] Salle H Pocard M Lehmann-Che J Bourthoumieu S Labrousse F Pimpie C. Development of a novel orthotopic primary human chordoma xenograft model: A relevant support for future research on chordoma. J Neuropathol Exp Neurol (2020) 79:314–24. doi: 10.1093/jnen/nlz121 31841164

[32] Dobrolecki LE Airhart SD Alferez DG Aparicio S Behbod F Bentires-Alj M. Patient-derived xenograft (PDX) models in basic and translational breast cancer research. Cancer Metastasis Rev (2016) 35:547–73. doi: 10.1007/s10555-016-9653-x PMC5396460 28025748

[33] Diaz RJ Luck A Bondoc A Golbourn B Picard D Remke M. Characterization of a clival chordoma xenograft model reveals tumor genomic instability. Am J Pathol (2018) 188:2902–11. doi: 10.1016/j.ajpath.2018.08.004 30248342

[34] Zhao T Siu I-M Williamson T Zhang H Ji C Burger PC. AZD8055 enhances *in vivo* efficacy of afatinib in chordomas. J Pathol (2021). doi: 10.1002/path.5739 PMC9534552 34124783

[35] Passeri T Dahmani A Masliah-Planchon J Naguez A Michou M El Botty R. Dramatic *In vivo* efficacy of the EZH2-inhibitor tazemetostat in PBRM1-mutated human chordoma xenograft. Cancers (2022) 14:1486. doi: 10.3390/cancers14061486 35326637 PMC8946089

[36] Masliah-Planchon J Bièche I Guinebretière J-M Bourdeaut F Delattre O. SWI/SNF chromatin remodeling and human malignancies. Annu Rev Pathol Mech Dis (2015) 10:145–71. doi: 10.1146/annurev-pathol-012414-040445 25387058

[37] Duan R Du W Guo W. EZH2: A novel target for cancer treatment. J Hematol Oncol (2020) 13:104. doi: 10.1186/s13045-020-00937-8 32723346 PMC7385862

[38] Gounder MM Zhu G Roshal L Lis E Daigle SR Blakemore SJ. Immunologic correlates of the abscopal effect in a SMARCB1/INI1-negative poorly differentiated chordoma after EZH2 inhibition and radiotherapy. Clin Cancer Res (2019) 25:2064–71. doi: 10.1158/1078-0432.CCR-18-3133 PMC9165752 30642912

[39] Witkiewicz AK Knudsen KE Dicker AP Knudsen ES. The meaning of p16 ^ink4a^ expression in tumors: Functional significance, clinical associations and future developments. Cell Cycle (2011) 10:2497–503. doi: 10.4161/cc.10.15.16776 PMC3685613 21775818

[40] Nobori T Miura K Wu DJ Lois A Takabayashi K Carson DA. Deletions of the cyclin-dependent kinase-4 inhibitor gene in multiple human cancers. Nature (1994) 368:753–6. doi: 10.1038/368753a0 8152487

[41] von Witzleben A Goerttler LT Marienfeld R Barth H Lechel A Mellert K. Preclinical characterization of novel chordoma cell systems and their targeting by pharmocological inhibitors of the CDK4/6 cell-cycle pathway. Cancer Res (2015) 75:3823–31. doi: 10.1158/0008-5472.CAN-14-3270 26183925

[42] Yuan K Wang X Dong H Min W Hao H Yang P. Selective inhibition of CDK4/6: A safe and effective strategy for developing anticancer drugs. Acta Pharm Sin B (2021) 11:30–54. doi: 10.1016/j.apsb.2020.05.001 33532179 PMC7838032

[43] Mayer EL Dueck AC Martin M Rubovszky G Burstein HJ Bellet-Ezquerra M. Palbociclib with adjuvant endocrine therapy in early breast cancer (PALLAS): Interim analysis of a multicentre, open-label, randomised, phase 3 study. Lancet Oncol (2021) 22:212–22. doi: 10.1016/S1470-2045(20)30642-2 33460574

[44] Gjertsen BT Schöffski P. Discovery and development of the polo-like kinase inhibitor volasertib in cancer therapy. Leukemia (2015) 29:11–9. doi: 10.1038/leu.2014.222 PMC4335352 25027517

[45] Steegmaier M Hoffmann M Baum A Lénárt P Petronczki M Krssák M. BI 2536, a potent and selective inhibitor of polo-like kinase 1, inhibits tumor growth. vivo. Curr Biol (2007) 17:316–22. doi: 10.1016/j.cub.2006.12.037 17291758

[46] Montaudon E El Botty R Vacher S Déas O Naguez A Chateau-Joubert S. High *in vitro* and *in vivo* synergistic activity between mTORC1 and PLK1 inhibition in adenocarcinoma NSCLC. Oncotarget (2021) 12:859–72. doi: 10.18632/oncotarget.27930 PMC8057272 33889306

[47] Montaudon E Nikitorowicz-Buniak J Sourd L Morisset L El Botty R Huguet L. PLK1 inhibition exhibits strong anti-tumoral activity in CCND1 -driven breast cancer metastases with acquired palbociclib resistance. Nat Commun (2020) 11:4053. doi: 10.1038/s41467-020-17697-1 32792481 PMC7426966

[48] Barber SM Sadrameli SS Lee JJ Fridley JS Teh BS Oyelese AA. Chordoma–current understanding and modern treatment paradigms. JCM (2021) 10:1054. doi: 10.3390/jcm10051054 33806339 PMC7961966

[49] Johnson JI Decker S Zaharevitz D Rubinstein LV Venditti JM Schepartz S. Relationships between drug activity in NCI preclinical *in vitro* and *in vivo* models and early clinical trials. Br J Cancer (2001) 84:1424–31. doi: 10.1054/bjoc.2001.1796 PMC2363645 11355958

[50] Bièche I Parfait B Tozlu S Lidereau R Vidaud M. Quantitation of androgen receptor gene expression in sporadic breast tumors by real-time RT-PCR: evidence that MYC is an AR-regulated gene. Carcinogenesis (2001) 22:1521–6. doi: 10.1093/carcin/22.9.1521 11532875

[51] Pandey K An H Kim SK Lee SA Kim S Lim SM. Molecular mechanisms of resistance to CDK4/6 inhibitors in breast cancer: A review. Int J Cancer (2019) 145:1179–88. doi: 10.1002/ijc.32020 PMC6767051 30478914

[52] Li Z Zou W Zhang J Zhang Y Xu Q Li S. Mechanisms of CDK4/6 inhibitor resistance in luminal breast cancer. Front Pharmacol (2020) 11:580251. doi: 10.3389/fphar.2020.580251 33364954 PMC7751736

[53] Rubino F Alvarez-Breckenridge C Akdemir K Conley AP Bishop AJ Wang W-L. Prognostic molecular biomarkers in chordomas: A systematic review and identification of clinically usable biomarker panels. Front Oncol (2022) 12:997506. doi: 10.3389/fonc.2022.997506 36248987 PMC9557284

[54] Al Baghdadi T Halabi S Garrett-Mayer E Mangat PK Ahn ER Sahai V. Palbociclib in patients with pancreatic and biliary cancer with CDKN2A alterations: Results from the targeted agent and profiling utilization registry study. JCO Precis Oncol (2019), 1–8. doi: 10.1200/PO.19.00124 35100714

[55] Xue Z Lui VWY Li Y Jia L You C Li X. Therapeutic evaluation of palbociclib and its compatibility with other chemotherapies for primary and recurrent nasopharyngeal carcinoma. J Exp Clin Cancer Res (2020) 39:262. doi: 10.1186/s13046-020-01763-z 33243298 PMC7690146

[56] Anderson E Havener TM Zorn KM Foil DH Lane TR Capuzzi SJ. Synergistic drug combinations and machine learning for drug repurposing in chordoma. Sci Rep (2020) 10:12982. doi: 10.1038/s41598-020-70026-w 32737414 PMC7395084

[57] Gill CM Fowkes M Shrivastava RK. Emerging therapeutic targets in chordomas: A review of the literature in the genomic era. Neurosurgery (2019). doi: 10.1093/neuros/nyz342 31504814

[58] El-Heliebi A Kroneis T Wagner K Meditz K Kolb D Feichtinger J. Resolving tumor heterogeneity: Genes involved in chordoma cell development identified by low-template analysis of morphologically distinct cells. PloS One (2014) 9:e87663. doi: 10.1371/journal.pone.0087663 24503940 PMC3913634

[59] Zhang J Späth SS Marjani SL Zhang W Pan X. Characterization of cancer genomic heterogeneity by next-generation sequencing advances precision medicine in cancer treatment. Precis Clin Med (2018) 1:29–48. doi: 10.1093/pcmedi/pby007 30687561 PMC6333046

[60] Wander SA Cohen O Gong X Johnson GN Buendia-Buendia JE Lloyd MR. The genomic landscape of intrinsic and acquired resistance to cyclin-dependent kinase 4/6 inhibitors in patients with hormone receptor–positive metastatic breast cancer. Cancer Discovery (2020) 10:1174–93. doi: 10.1158/2159-8290.CD-19-1390 PMC8815415 32404308

[61] Hanahan D Weinberg RA. Hallmarks of cancer: The next generation. Cell (2011) 144:646–74. doi: 10.1016/j.cell.2011.02.013 21376230

[62] Warburg O. On respiratory impairment in cancer cells. Science (1956) 124:269–70. doi: 10.1126/science.124.3215.269 13351639

[63] Lorito N Bacci M Smiriglia A Mannelli M Parri M Comito G. Glucose metabolic reprogramming of ER breast cancer in acquired resistance to the CDK4/6 inhibitor palbociclib+. Cells (2020) 9:668. doi: 10.3390/cells9030668 32164162 PMC7140692

[64] Zaal EA Berkers CR. The influence of metabolism on drug response in cancer. Front Oncol (2018) 8:500. doi: 10.3389/fonc.2018.00500 30456204 PMC6230982

[65] Pavlides S Whitaker-Menezes D Castello-Cros R Flomenberg N Witkiewicz AK Frank PG. The reverse warburg effect: Aerobic glycolysis in cancer associated fibroblasts and the tumor stroma. Cell Cycle (2009) 8:3984–4001. doi: 10.4161/cc.8.23.10238 19923890

[66] Evans KW Yuca E Scott SS Zhao M Paez Arango N Cruz Pico CX. Oxidative phosphorylation is a metabolic vulnerability in chemotherapy-resistant triple negative breast cancer. Cancer Res (2021). doi: 10.1158/0008-5472.CAN-20-3242 PMC8563442 34518211

[67] Chen D Barsoumian HB Fischer G Yang L Verma V Younes AI. Combination treatment with radiotherapy and a novel oxidative phosphorylation inhibitor overcomes PD-1 resistance and enhances antitumor immunity. J Immunother Cancer (2020) 8:e000289. doi: 10.1136/jitc-2019-000289 32581056 PMC7319777

[68] Lee J-S Lee H Jang H Woo SM Park JB Lee S-H. Targeting oxidative phosphorylation reverses drug resistance in cancer cells by blocking autophagy recycling. Cells (2020) 9:2013. doi: 10.3390/cells9092013 32883024 PMC7565066

[69] Sica V Bravo-San Pedro JM Stoll G Kroemer G. Oxidative phosphorylation as a potential therapeutic target for cancer therapy. Int J Cancer (2020) 146:10–7. doi: 10.1002/ijc.32616 31396957

[70] Teh JLF Purwin TJ Han A Chua V Patel P Baqai U. Metabolic adaptations to MEK and CDK4/6 cotargeting in uveal melanoma. Mol Cancer Ther (2020) 19:1719–26. doi: 10.1158/1535-7163.MCT-19-1016 PMC7415561 32430489

[71] Posch C Cholewa BD Vujic I Sanlorenzo M Ma J Kim ST. Combined inhibition of MEK and Plk1 has synergistic antitumor activity in NRAS mutant melanoma. J Invest Dermatol (2015) 135:2475–83. doi: 10.1038/jid.2015.198 PMC4567913 26016894

[72] Gutteridge REA Ndiaye MA Liu X Ahmad N. Plk1 inhibitors in cancer therapy: From laboratory to clinics. Mol Cancer Ther (2016) 15:1427–35. doi: 10.1158/1535-7163.MCT-15-0897 PMC4936921 27330107

